# Butyrate interacts with the effects of 2’FL and 3FL to modulate *in vitro* ovalbumin-induced immune activation, and 2’FL lowers mucosal mast cell activation in a preclinical model for hen’s egg allergy

**DOI:** 10.3389/fnut.2023.1305833

**Published:** 2023-12-19

**Authors:** M. Zuurveld, M. A. P. Diks, P. C. J. Kiliaan, J. Garssen, G. Folkerts, B. van’t Land, L. E. M. Willemsen

**Affiliations:** ^1^Division of Pharmacology, Faculty of Science, Utrecht Institute for Pharmaceutical Sciences, Utrecht University, Utrecht, Netherlands; ^2^Danone Nutricia Research B.V, Utrecht, Netherlands; ^3^Center for Translational Immunology, University Medical Center Utrecht, Utrecht, Netherlands

**Keywords:** allergic sensitization, human milk oligosaccharides, mucosal immunology, short-chain fatty acids, food allergy

## Abstract

**Background:**

Early life provides a window of opportunity to prevent allergic diseases. With a prevalence of 0.5–2% in infants, hen’s egg allergy is one of the most common food allergies. The immunomodulatory effects of human milk oligosaccharides (HMOs), 2′-fucosyllactose (2’FL), and 3-fucosyllactose (3FL) were studied in an *in vitro* mucosal immune model and an *in vivo* murine model for hen’s egg (ovalbumin) allergy.

**Methods:**

Intestinal epithelial cell (IEC)/dendritic cell (DC) and DC/T cell cocultures were used to expose IECs to ovalbumin (OVA) in an *in vitro* mucosal immune model. The effects of epithelial pre-incubation with 0.1% 2’FL or 3FL and/or 0.5 mM butyrate were studied. Three- to four-weeks-old female C3H/HeOuJ mice were fed AIN93G diets containing 0.1–0.5% 2’FL or 3FL 2 weeks before and during OVA sensitization and challenge. Allergic symptoms and systemic and local immune parameters were assessed.

**Results:**

Exposing IECs to butyrate *in vitro* left the IEC/DC/T cell cross-talk unaffected, while 2’FL and 3FL showed differential immunomodulatory effects. In 3FL exposed IEC-DC-T cells, the secretion of IFNγ and IL10 was enhanced. This was observed upon pre-incubation of IECs with 2’FL and butyrate as well, but not 2’FL alone. The presence of butyrate did not affect OVA activation, but when combined with 3FL, an increase in IL6 release from DCs was observed (*p* < 0.001). OVA allergic mice receiving 0.5% 3FL diet had a lower %Th2 cells in MLNs, but the humoral response was unaltered compared to control mice. OVA-allergic mice receiving 0.1 or 0.5% 2’FL diets had lower serum levels of OVA-IgG2a (*p* < 0.05) or the mast cell marker mMCP1, in association with increased concentration of cecal short-chain fatty acids (SCFAs) (*p* < 0.05).

**Conclusion:**

*In vitro* butyrate exposure promotes the development of a downstream type 1 and regulatory response observed after 2’FL exposure. 2’FL and 3FL differentially modulate ovalbumin-induced mucosal inflammation predominantly independent of butyrate. Mice receiving dietary 3FL during ovalbumin sensitization and challenge had lowered Th2 activation while the frequency of Treg cells was enhanced. By contrast, 2’FL improved the humoral immune response and suppressed mast cell activation in association with increased SCFAs production in the murine model for hen’s egg allergy.

## 1 Introduction

Short-chain fatty acids (SCFAs) are known for their immunomodulatory effects and are formed during fermentation of non-digestible oligosaccharides (NDOs) by the intestinal microbiota. In early life, human milk oligosaccharides (HMOs), present in breast milk, form the major source of NDOs. Various intestinal microbial species are equipped to metabolize HMOs into SCFAs (1, 2). Immunomodulatory properties have been attributed to HMOs and SCFAs, including both direct effects on immune cells and indirect effects via the microbiome (3). Some of these effects involve promoted mucus production, improved gut integrity, interaction with G protein-coupled receptors (GPRs), inhibition of histone deacetylases (HDACs), and the NF-κB pathway in intestinal epithelial cells (IECs), as well as improved Treg formation (4–9). Many of these pathways are involved in allergic diseases also.

An increasing percentage of the human population is developing allergic diseases, especially early in life (10). Belonging to the top three most common food allergens, 0.5 to 2% of infants become allergic to hen’s egg, of which ovalbumin is one of the major allergens. Ovalbumin (OVA), also known as Gal d2, is a phosphoglycoprotein with a molecular weight of ~44.5 kDa (11). As curative treatments for allergic diseases are not available yet, the focus has shifted to studying potential preventive strategies. Early life has been identified as a window of opportunity for preventing food allergic diseases. During the first 1,000 days of life, the immune system matures and moves away from the pre- and neonatal-associated Th2-dominated immune status, while the microbiome develops into an adult-like steady state (12–14).

Human milk contains very specific structures and uniquely high levels of oligosaccharides, indicating that these structures may have a biological function during the development of neonates (15). Depending on, e.g., the genetic background and stage of lactation, the mother mainly excretes 2′-fucosyllactose (2’FL) or 3-fucosyllactose (3FL) (16, 17). Previously, we showed that *in vitro* exposure to 2’FL and 3FL resulted in a differential immunomodulatory effect during *in vitro* OVA-induced mucosal inflammation (18). Specific bifidobacteria and lactobacilli can metabolize fucosylated HMOs into SCFAs (19). SCFAs, in particular butyrate, are considered to possess the most potent immunomodulatory properties (20) and restore or maintain homeostasis in mucosal immune responses (21–24). Furthermore, neonates with high butyrate levels in fecal samples were less likely to develop allergic rhinitis, allergic asthma, and food allergy during childhood (25), substantiating the potential protective role of butyrate in the development of allergic sensitization.

To the best of our knowledge, interactions between HMOs and SCFAs have not been studied so far *in vitro*, while the exposure occurs simultaneously *in vivo*. Therefore, this study investigated the potential interaction between butyrate and the most common fucosylated human milk oligosaccharides 2’FL and 3FL on intestinal epithelial function during homeostasis or upon OVA-induced type 2 activation and subsequent dendritic cell maturation and T cell functioning in a mucosal coculture model. To validate these findings, 2’FL or 3FL supplemented diets were applied in a murine model for OVA-induced food allergy. Furthermore, systemic allergic symptoms after challenge, as well as additional immunological parameters and SCFA levels in the cecum, were determined.

## 2 Materials and methods

### 2.1 HT-29 cell culturing

The human colon adenocarcinoma HT-29 cell line (passages 148–156) was used to model IEC. The cells were cultured in McCoy’s 5A medium (Gibco, United States) supplemented with 10% fetal calf serum (Gibco), penicillin (100 U/mL), and streptomycin (100 μg/mL) (Sigma-Aldrich, United Kingdom). The IEC were cultured at 5% CO2 influx and 37°C. Medium refreshments occurred every 2–3 days, and the cells were trypsinized at 80–90% confluency to passage. Cells were diluted 6–8 times based on surface coverage upon transfer to 12-well transwell plates (Corning Costar, United States). After 5 days in the transwell, the cells had grown confluent and were used for experimental exposures.

### 2.2 PBMC, monocyte, and naïve T cell isolation and culture

Isolation of human PBMCs from buffy coats from healthy donors (Dutch Blood Bank, Netherlands) was performed by collecting the enriched fraction of cells after density gradient centrifugation (Greiner Bio-One, The Netherlands) as previously described (18). Monocytes were isolated from PBMCs through magnetic separation via negative selection according to the manufacturer’s instructions (Miltenyi Biotec, Germany). Subsequently, monocytes were cultured for 7 days in RPMI 1640 (Lonza, Switzerland) with 10% FCS, penicillin (100 U/mL), and streptomycin (100 μg/mL) at 2×10^6 cells/mL. Human recombinant IL4 (100 ng/mL) and GM-CSF (60 ng/mL) (Prospec, Israel) were added to differentiate monocytes into monocyte-derived dendritic cells (moDCs). Every other day half of the medium was refreshed and new cytokines were added until the moDCs were collected for coculture. Naïve CD4+ T cells were isolated from PBMCs using a negative selection MACS kit according to the manufacturer’s instructions (Miltenyi Biotec). The collected T cells were frozen in 90% FCS and 10% DMSO at −80°C until use during coculture. Upon gentle thawing, the cells were resuspended in T cell medium (IMDM with 10% FCS, penicillin (100 U/mL), streptomycin (100 μg/mL), 20 μg/mL apotransferrin (Sigma-Aldrich), and 50 μM β-mercaptoethanol) and directly used.

### 2.3 IEC, IEC-moDC, and moDC-T cell model description

This model is previously described (18) and can be used to study the cross-talk between epithelial cells and dendritic cells, and subsequently dendritic cells and naïve T cells during allergic sensitization.

**Figure 1:**
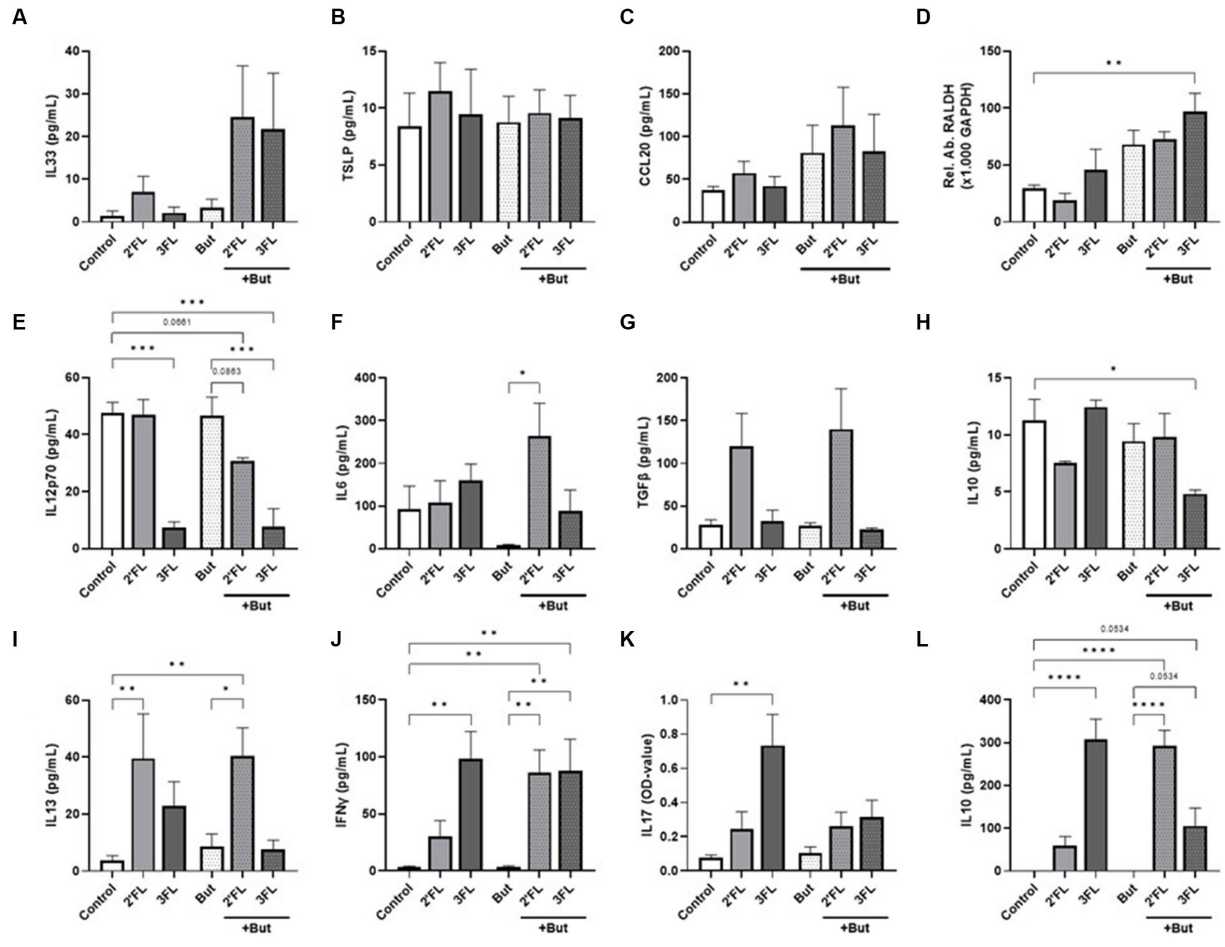
Cytokine and chemokine release from IEC, IEC/moDC, and moDC/T cell cocultures after 48 h incubation of IEC with butyrate and/or 2’FL or 3FL. IEC supernatant concentrations of **(A)** IL33, **(B)** TSLP, **(C)** CCL20, and **(D)** relative abundance of RALDH mRNA were measured. Secretion of **(E)** IL12p70, **(F)** IL6, **(G)** TGFβ, and **(H)** IL10 was assessed after IEC/moDC coculture. Secretion of the functional cytokines **(I)** IL13, **(J)** IFNγ, **(K)** IL17, and **(L)** IL10 after moDC/T cell coculture were measured. Data were analyzed by one-way ANOVA followed by Bonferroni’s post-hoc test, *n* = 3–6, mean ± SEM (*p < 0.05, **p < 0.01, ***p < 0.001, ****p < 0.0001).

**Figure 2:**
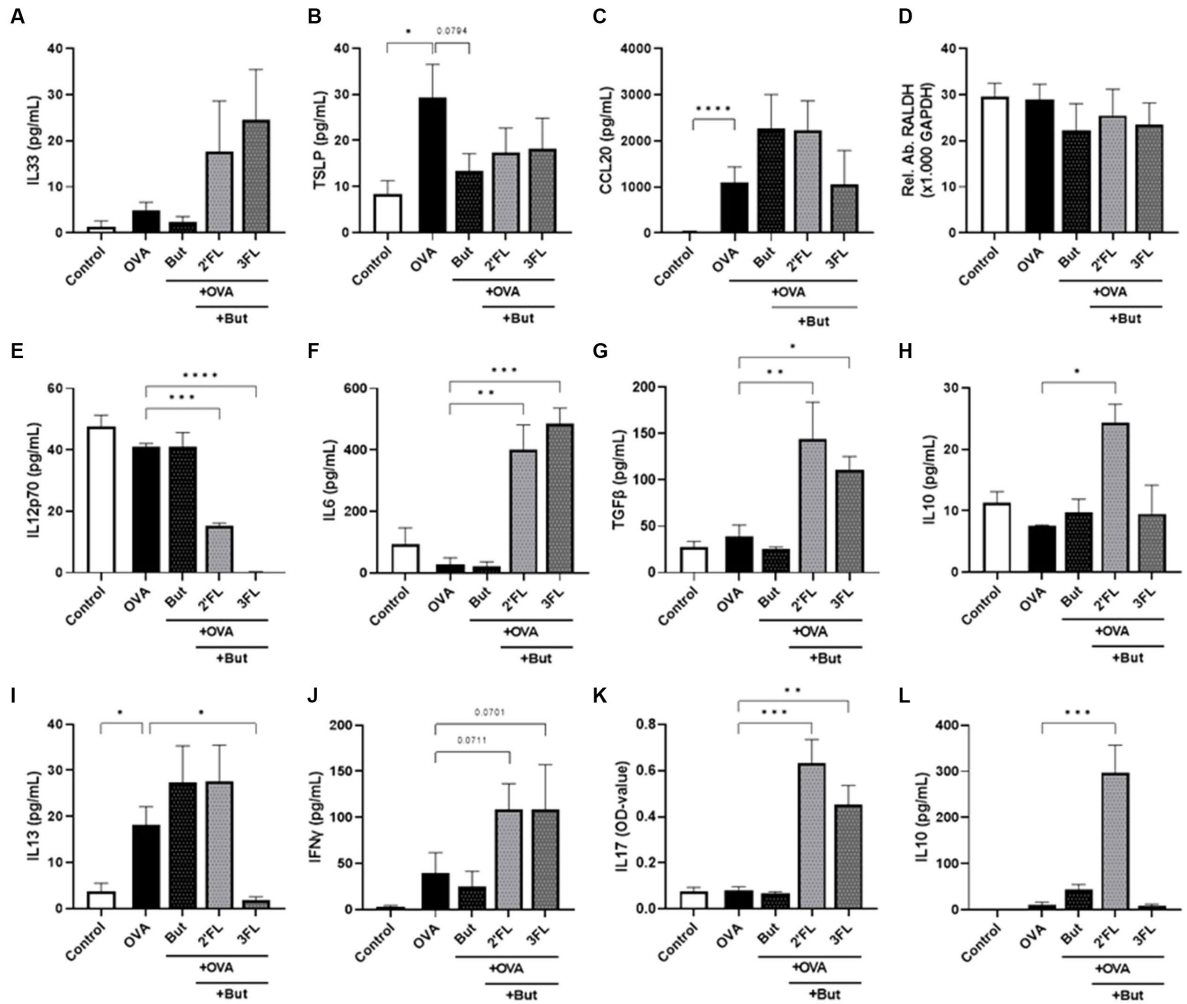
Cytokine and chemokine release from IEC, IEC/moDC, and moDC/T cell cocultures after 24 h incubation of IEC with butyrate and/or 2’FL or 3FL and subsequent 24 h exposure to OVA. IEC supernatant concentrations of **(A)** IL33, **(B)** TSLP, **(C)** CCL20, and **(D)** relative abundance of RALDH mRNA were measured. The phenotype of moDCs and mediator secretion was assessed after IEC/moDC coculture. Secretion of **(E)** IL12p70, **(F)** IL6, **(G)** TGFβ, and **(H)** IL10 was assessed after IEC/moDC coculture. Secretion of the functional cytokines **(I)** IL13, **(J)** IFNγ, **(K)** IL17, and **(L)** IL10 after moDC/T cell coculture were measured. Data were analyzed by one-way ANOVA followed by Dunnett’s post-hoc test as all groups were compared to the OVA group, *n* = 3–6, mean ± SEM (*p < 0.05, **p < 0.01, ***p < 0.001, ****p < 0.0001).

Sodium butyrate (Sigma-Aldrich) was dissolved in PBS (0.5 M). The 2’FL and 3FL, enzymatically produced from lactose, were purchased from Carbosynth (UK) and dissolved in PBS (10 mg/mL, 10% w/v%). HMOs and butyrate solutions were sterile filtered (0.2 μm) before use. In short, HT29 cells cultured confluent in transwell plates were incubated with 2’FL or 3FL (0.1% w/v dissolved in McCoy’s 5A medium) (Carbosynth, United Kingdom) and/or butyrate (0.5 mM) (Sigma-Aldrich) for 24 h (butyrate concentration is based on data shown in Supplementary Figure S1). Thereafter, the medium was refreshed containing new HMOs and butyrate (Figure 1), and designated conditions were exposed to ovalbumin (100 μg/mL, Sigma-Aldrich) for another 24 h (Figure 2). The basolateral medium was collected for cytokine analysis by ELISA, and after washing the HT29 cells with fresh medium, moDCs were added to the basolateral compartment for 48 h. Upon coculture, moDC were collected for FACS analysis or subsequent coculture with naïve T cells for 5 days [1:10 ratio, in the presence of 150 ng/mL anti-CD3 (Sanquin, Netherlands) and 5 ng/mL IL2 (Prospec, Israel)]. Control and OVA conditions in Figure 2 are shared with a previous dataset (18).

### 2.4 Animals

Three- to four-week-old female C3H/HeOuJ mice were purchased from Charles River (Germany) and were randomly assigned to the control (*n* = 6) or experimental groups (*n* = 12). The mice were housed under sterile conditions (2–4 cages/group) with standard chip bedding, tissues, and a plastic shelter on a 12 h light/dark cycle with controlled temperature and humidity. The mice had *ad libitum* access to the AIN93-G diet supplemented with or without 0.1% or 0.5% of 2’FL or 3FL (2’FL and 3FL were purchased from Jennewein Biotechnologie GmbH, Germany, and the diets were produced by Sniff Spezialdiëten GMBH, Germany) and water. Supplementation of intervention diets with 2’FL or 3FL was isocaloric compensated with the reduction in cellulose. Animal procedures were conducted in accordance with the Animal Welfare Body according to institutional guidelines for the care and use of laboratory animals as established by the Animal Ethics Committee of Utrecht University (AVD108002015262).

### 2.5 Animal procedures

**Figure 3:**
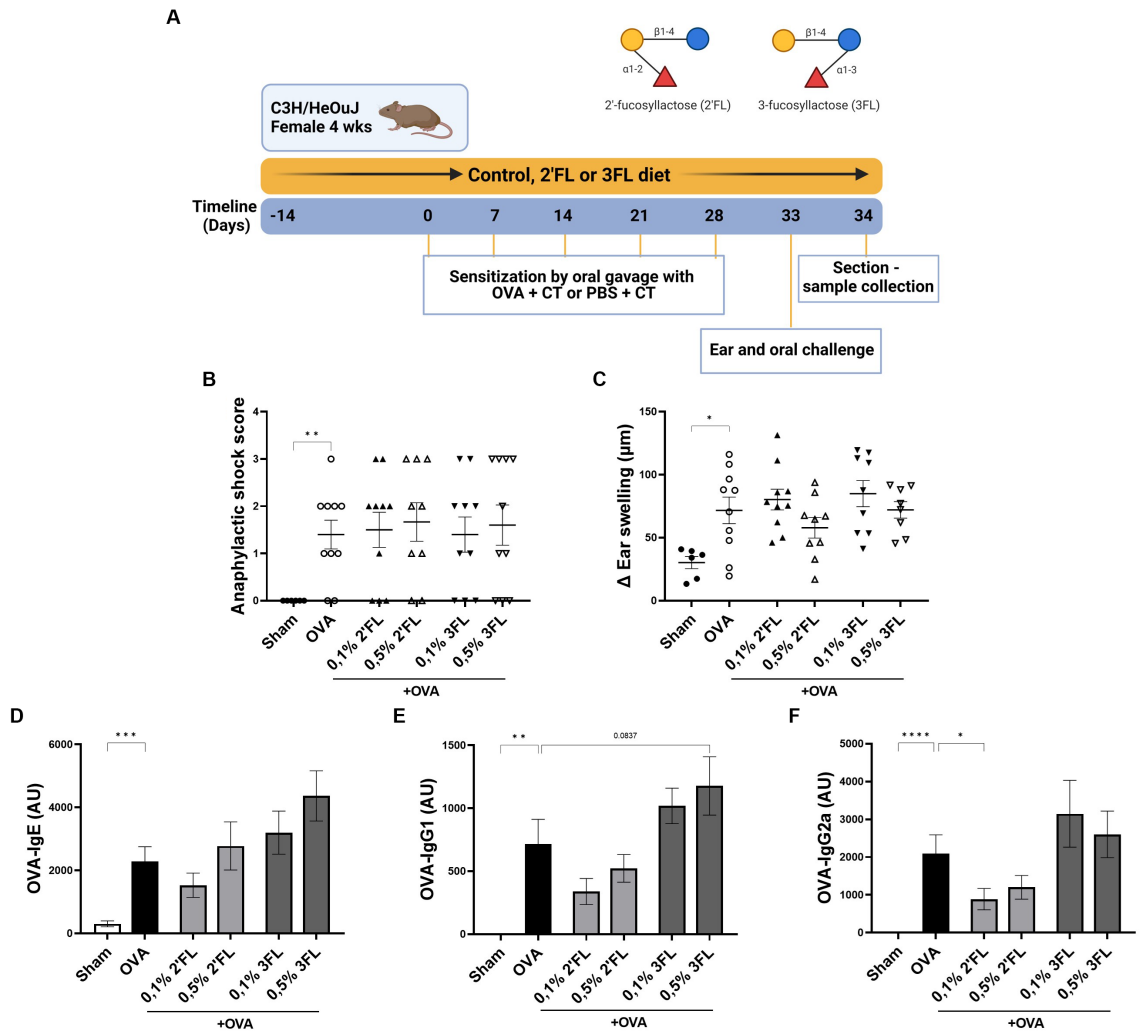
The effects of dietary 2’FL and 3FL intervention on OVA food allergy in mice on anaphylactic shock and serum immunoglobulins. **(A)** The design of the study is shown. **(B)** The anaphylactic shock score and **(C)** the increase in ear thickness 1 h after intradermal challenge are shown. Relative levels of **(D)** OVA-specific IgE, **(E)** IgG1, and **(F)** IgG2a were measured in serum 18 h after oral challenge. Sham and OVA groups were analyzed by an unpaired *t*-test. All dietary intervention groups were compared to the OVA group using a one-way ANOVA followed by Dunnett’s post-hoc test, *n* = 6–12, mean ± SEM (*p < 0.05, **p < 0.01, ***p < 0.001, ****p < 0.0001).

A schematic overview of the experimental design is given in Figure 3A. Mice received the HMOs supplemented or control diets 2 weeks before the first sensitization. Oral sensitization occurred on experimental days 0, 7, 14, 21, and 28 with 20 mg ovalbumin (Grade V; Sigma Aldrich, United States) and 10 μg cholera toxin (List Biological Laboratories, United States) in 500 μL sterile PBS by oral gavage. Sham mice (*n* = 6) received cholera toxin in 500 μL sterile PBS alone. Five days after the final sensitization, mice were intradermally challenged in both ears with 12.5 μg OVA and 25 μL PBS to determine acute allergic skin and systemic shock response. Six hours after the intradermal challenge, mice were orally challenged with 50 mg OVA in 500 μL PBS. Eighteen hours later, the mice were sacrificed, and tissue samples were collected.

### 2.6 Assessment of clinical symptoms

To evaluate the severity of the allergic response to OVA, mice were intradermally challenged in both ears. Acute allergic skin response, anaphylactic shock symptoms, and body temperature were determined by individuals blinded to the intervention groups. The acute allergic skin response was determined by measuring the ear thickness of anesthetized mice before and 1 h after intradermal challenge, subtracting the basal ear thickness from the ear thickness 1 h after intradermal challenge results in the Δ ear swelling (μm).

### 2.7 cDNA synthesis and real-time qPCR

HT29 cells were cultured in 48 well plates for 6 days and pre-incubated for 24 h with butyrate and/or 2’FL or 3FL before OVA exposure for 24 h. HT29 cells were lysed in RNA lysis buffer (provided with an RNA isolation kit) and stored at −70°C. Total mRNA content was isolated, cDNA was synthesized, and real-time PCR was performed as previously described (18). The relative expression of the gene of interest over housekeeping gene GAPDH was calculated: relative mRNA abundance = 100.000× 2 Ct[GAPDH mRNA] – Ct[target mRNA] (26).

### 2.8 Enzyme-linked immunosorbent assay

Supernatants collected from the different cell culture steps were analyzed for chemokine and cytokine secretion with enzyme-linked immunosorbent assays. ELISA kits were used to determine IL6, IL10, IL13, IL17, IFNγ, TGFβ, TSLP (Thermo Fischer scientific, United States), CCL20, IL12p70, and IL33 (R&D systems, United States) secretion following the manufacturer’s protocols.

OVA-specific immunoglobulins were measured in murine serum. Serial dilutions of pooled serum were used to generate a standard curve. For OVA-specific IgE, 96-well high-binding plates (Costar Corning Incorporated, United States) were coated with 100 μL rat anti-mouse IgE (2 μg/mL in PBS; BD Biosciences, United States) overnight and washed. After blocking (50 mM Tris, 2 mM EDTA, 136.9 mM NaCl, 0.05% Tween20, and 1% BSA), diluted serum samples were incubated for 2 h. After washing (PBS, 0.05% Tween), 100 μL Biotinylated OVA (1 μg/mL) was added for 90 min. For the latter purpose, OVA (Sigma-Aldrich, Netherlands) was biotinylated using an EZ-Link™ Sulfo-NHS-LC-Biotinylation kit (ThermoFisher Scientific, United States) according to the manufacturer’s instructions. Plates were washed and incubated with streptavidin-HRP (Sanquin, The Netherlands). To measure OVA-specific IgG1 and IgG2a, 96-well high-binding plates (Costar Corning Incorporated, United States) were coated with 100 μL OVA (10 μg/mL in PBS) overnight. After blocking, diluted samples were incubated for 2 h. After washing, 100 μL biotinylated anti-mouse IgG1 or anti-mouse IgG2a (BD Biosciences) was added for 90 min, and plates were washed and incubated with streptavidin-HRP. The colorimetric reaction was started by the addition of 100 μL o-phenylenediamine dihydrochloride (0.4 mg/mL; Sigma-Aldrich) and the reaction was stopped by 75 μL of 4 M H_2_SO_4_.

### 2.9 Flow cytometry

All cells collected for flow cytometric analysis were transferred to 96-well plates (Costar Corning Incorporated). After washing the cells with PBS, the viability of the cells was determined with Fixable Viability Dye 780-APC Cyanine 7 (eBioscience). Blocking buffer [PBS with 2.5% FCS and human Fc block (BD Biosciences, United States)] or FcR blocking reagent mouse (Miltenyi Biotech, United States) was added for 15 min at 4°C to prevent non-specific binding of antibodies. Murine samples were stained using titrated volumes of the following antibodies: CD4-BV510 (clone RM4-5), CD69-PE-Cy7 (clone H1.2F3), CXCR3-PE (clone CXCR3 473), T1ST2-FITC (clone DJ8), CD25-PerCP-Cy5.5 (clone 3C7), and FoxP3-FITC (clone FJK-16 s). After 30 min of staining at 4°C, stained cells were washed. Cells were resuspended, flow cytometric measurements were performed using BD FACS Canto II (Becton Dickinson, United States), and acquired data were analyzed using FlowLogic software (Inivai Technologies, Australia). A representative gating strategy is shown in Supplementary Figure S2.

### 2.10 Short-chain fatty acids detection

Cecum contents were diluted five times in ice-cold PBS. Then, 10 to 20 1.0 mm glass beads (BioSpec, United States) were added. Samples were homogenized on a vortex for 90 s. After centrifugation for 10 min (13.000 RPM) at 4°C, supernatants were collected and stored at −80°C until further analysis. SCFA levels were detected by gas chromatography (Shimadzu GC2010, Shimadzu Corporation, Japan).

### 2.11 Statistical analysis

Statistical analyses were performed using GraphPad Prism (Version 9.4.1) software.

*In vitro* data were analyzed by one-way ANOVA followed by Bonferroni’s post-hoc test on selected pairwise comparisons or Dunnett’s multiple comparisons test when all conditions were compared to the OVA-exposed condition.

*In vivo* data were analyzed using an unpaired *t*-test to compare Sham and OVA groups. All intervention groups were compared to the OVA group using one-way ANOVA followed by Dunnett’s multiple comparisons test. For SCFA analysis, all intervention groups were compared to the OVA group, and 0.1% 2’FL was compared to 0.1% 3FL as well as 0.5% 2’FL to 0.5% 3FL; therefore, the one-way ANOVA was followed by a Bonferroni’s post-hoc test with selected pairs.

If data did not fit a normal distribution, logarithmic transformation was applied before further analysis; *p* < 0.05 is considered statistically significant, and data are represented as mean + SEM of *n* = 6–10 animals per group or *n* = 3–6 independent paired *in vitro* repeats.

## 3 Results

### 3.1 Butyrate alone does not influence IEC/DC/T cell cross-talk, while immunomodulation by 2’FL and 3FL during homeostasis is altered in the presence of butyrate

To investigate the potential interaction between butyrate exposure and two commonly expressed fucosylated HMOs, IECs were cultured in transwells and apically incubated for 48 h with butyrate (0.5 mM) and/or 2’FL or 3FL (0.1% w/v). After incubation, IECs were washed and cocultured with moDCs for 2 days. The primed moDCs were cocultured with naïve T cells to assess functional immune outcomes. See the Glossary for a short description of the indicated models and abbreviations.

Exposing IEC to butyrate (but-IEC) or HMOs (2’FL-IEC or 3FL-IEC) did not affect IEC-derived IL33, TSLP, or IL25 secretion (Figures 1A–C) nor RALDH expression (Figure 1D). However, combined exposure of butyrate and 3FL (but-3FL-IEC) enhanced the relative expression of RALDH mRNA in IEC significantly compared to the control cells (Figure 1D). After washing the exposed IEC and subsequent coculture with moDCs (IEC/DC), mediator secretion from IEC/DC (Figures 1E–H) was measured. The mediator release was not significantly affected after the coculture of DC with either but-IEC or 2’FL-IEC. However, when butyrate incubation was combined with 2’FL (but-2’FL-IEC/DC), IL12p70 secretion tended to decrease (Figure 1E), while IL6 secretion increased (Figure 1F) as compared to moDC coculture with but-IEC. These data indicate that combined exposure of IEC to 2’FL and butyrate results in a different functional DC phenotype upon coculture with these pre-exposed IEC, compared to exposing the IEC to 2’FL or butyrate separately. Similar to the combination of 2’FL and butyrate, 3FL exposure alone already inhibited IL12p70 secretion (Figure 1E), which remained stable when combined with butyrate (but-3FL-IEC/DC). But-3FL-IEC/DC reduced the secretion of regulatory IL10 compared to control (Figure 1H). Although TGFβ is known as an important regulator in mucosal immunity, none of the incubations significantly affected secretion of TGFβ (Figure 1G).

To study the functional outcomes of the differentially primed moDCs, these cells were cocultured with naïve T cells (IEC/DC/T cells). The T cell cytokine secretion was not affected by coculture with primed DC derived from but-IEC/DC compared to control (Figures 1I–L). Coculture with 2’FL-IEC/DCs enhanced the secretion of IL13 in T cells (Figure 1I); in contrast, 3FL-IEC/DC/T cells enhanced the secretion of IFNγ, IL17, and IL10 (Figures 1J–L). But-2’FL-IEC/DC/T still showed increased IL13 secretion; yet, in addition, enhanced secretion of IFNγ and IL10 was observed as compared to control and butyrate alone. Furthermore, but-3FL-IEC/DC/T cells were found to maintain the enhanced secretion of IFNγ and IL10 (*p* = 0.0534), while the IL17 secretion remained low compared to control and butyrate alone (Figures 1K,L).

### 3.2 Butyrate supports 3FL-mediated IL6 release from DCs and IFNγ secretion from T cells in an OVA-induced mucosal type 2 inflammation model

To assess the impact of HMOs and interaction with butyrate in an inflammatory condition, we aimed to study the effects of butyrate and/or 2’FL or 3FL in a model of OVA-induced epithelial inflammation. This model was recently used to demonstrate the differential immunomodulatory effects of 2’FL and 3FL (18); a summary of these results is displayed in Table 1, and a heatmap of the outcomes is given in Supplementary Figure S3. Incubating IEC with butyrate before OVA (but-OVA-IEC) exposure tended to prevent OVA-mediated TSLP release (*p* = 0.0794, Figure 2B). The OVA-induced CCL20 release remained unaffected by exposure to butyrate and HMOs (Figure 2C). The other IEC-related mediators (IL33 and RALDH; Figures 2A,D) were also not affected by pre-incubation with butyrate and/or 2’FL or 3FL before OVA exposure. However, 2’FL pre-incubation of OVA-IEC alone resulted in enhanced RALDH mRNA levels (Table 1), which was prevented by combined incubation with butyrate (Figure 2D) as compared to OVA-exposed cells.

**Table 1:** Overview of mediator secretion (pg/mL unless indicated otherwise) and relative RALDH mRNA expression in the IEC-moDC-T cell model when IEC were exposed to 2’FL and 3FL prior to ovalbumin stimulation.

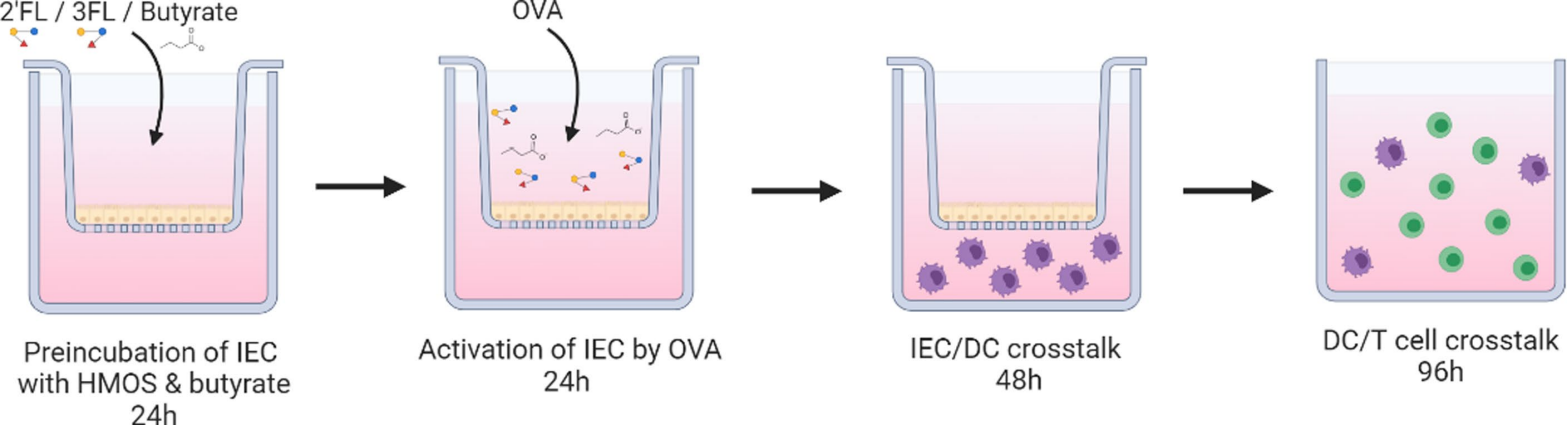				
	**Control**	**OVA**	**OVA + 2’FL**	**OVA + 3FL**
**IEC**				
IL33	1,4 ± 1,2	4,9 ± 1,7	11,5 ± 11,4	22,9 ± 9,6 ^*^
TSLP	8,4 ± 2,9	29,3 ± 7,2 ^*^	13,9 ± 3,1	17,5 ± 7,9
CCL20	37,2 ± 4,5	1088,0 ± 346,3 ^1^	2021,0 ± 701,4 ^**^	1217,0 ± 395,0 ^2^
RALDH (Rel. Ab.)	29,5 ± 3,0	28,9 ± 3,4	103,1 ± 11,7 ^***^	18,4 ± 1,7
**IEC/DC**				
IL12p70	47,5 ± 3,8	40,9 ± 1,2	30,3 ± 5,9 ^3^	4,9 ± 2,1 ^***^
IL6	92,3 ± 52,0	27,2 ± 20,9	240 ± 39	9,2 ± 3,1
TGFβ	27,5 ± 6,3	38,9 ± 22,0	51,5 ± 24,9	21,3 ± 2,0
IL10	11,3 ± 1,8	7,5 ± 0,2	13,7 ± 4,0	6,4 ± 2,2
**DC/T cell**				
IL13	3,6 ± 1,9	18,2 ± 4,0 ^**^	36,4 ± 10,2 ^**^	2,9 ± 1,6
IFNγ	3,3 ± 1,0	39,9 ± 21,7	109,8 ± 82,1 ^4^	67,9 ± 51,9
IL17 (OD-value)	0,07 ± 0,02	0,08 ± 0,02	0,6 ± 0,09 ^***^	0,7 ± 0,06 ^***^
IL10	1,5 ± 0,006	11,3 ± 5,1 ^**^	351,0 ± 54,0 ^****^	63,7 ± 18,6 ^***^

These data have previously been published (18). Data is analyzed by One-Way ANOVA followed by Dunnett’s post-hoc test compared to Control, *n* = 3–6, mean ± SEM (^1^ *p* = 0.0877, ^2^ *p* = 0.0528, ^3^ *p* = 0.0879, ^4^ *p* = 0.0628, *p < 0.05, **p < 0.01, ***p < 0.001, ****p < 0.0001).

After removal of butyrate, 2’FL, 3FL, and OVA from IEC by washing, the primed IEC were cocultured with moDCs. OVA exposure nor butyrate pre-incubation significantly affected the IEC/DC cytokine response (Figures 2E–H) compared to the control. Pre-incubation with 3FL, but not 2’FL, resulted in a lower IL12p70 secretion in OVA-IEC/DC (Table 1) compared to control, which was also observed in the absence of OVA (Figure 2E). Combined exposure to 2’FL and butyrate also resulted in lower levels of IL12p70 in OVA-IEC/DCs (but-2’FL-OVA-IEC/DCs), similar to but-3FL-OVA-IEC/DCs (Figure 2E), as compared to OVA-IEC/DC. IL6 secretion was enhanced after 2’FL pre-incubation of OVA-IEC/DCs (Table 1) and in but-2’FL-OVA-IEC/DCs (Figure 2F); this effect was observed also in the absence of OVA but to a much lesser extent (Figure 2G). Furthermore, TGFβ secretion was not affected by butyrate or 2’FL pre-incubation alone but was significantly increased in but-2’FL-OVA-IEC/DCs (Figure 2G). Interestingly, 3FL or butyrate pre-incubation separately did also not induce an increase in IL6 and TGFβ secretion by OVA-IEC/DCs (Table 1 and Figures 2F,G); however, enhanced IL6 and TGFβ secretion was measured in but-3FL-OVA-IEC/DCs similar to but-2’FL-OVA-IEC/DCs as compared to OVA-IEC/DCs. IL10 secretion was only significantly enhanced in but-2’FL-OVA-IEC/DCs compared to OVA-IEC/DCs (Figure 2H); this increase was not observed in the absence of OVA (Figure 2H) or butyrate (Table 1). This again shows the differential interaction between 2’FL and 3FL with butyrate, modifying the outcome of the IEC/DCs response.

After IEC/DC coculture, primed DCs were cocultured with allogenic naïve T helper cells to study functional immune outcomes. Although OVA-IEC/DC enhanced IL13 secretion during T cell coculture (Figure 2I) again, pre-incubation of OVA-IEC with butyrate alone had no significant effects on downstream OVA-induced T helper cell (but-OVA-IEC/DC/T cells) cytokine secretion (Figures 2I–L). 3FL pre-incubation prevented OVA-IEC/DC-mediated IL13 secretion by T cells (Table 1), which was also observed in but-3FL-OVA-IEC/DC/T cells (Figure 2I). The previously observed tendency to enhance IFNγ secretion in 2’FL-OVA-IEC/DC/T cells remained noticeable for but-2’FL-OVA-IEC/DC/T cells (*p* = 0.0711), similar to but-3FL-OVA-IEC/DC/T cells (*p* = 0.0701, Figure 2J) as compared to OVA-IEC/DC/T cells. These results were similar to the incubations in the absence of OVA (Figure 2J). IL17 levels were enhanced by 2’FL-OVA-IEC/DC/T cells and 3FL-OVA-IEC/DC/T cells (Table 1), which remained significantly enhanced in the presence of butyrate (Figure 2K) as compared to OVA-IEC/DC/T cells. In the absence of OVA, butyrate was able to dampen enhanced IL17 secretion after 3FL exposure (Figure 2K). Secretion of IL10 was significantly enhanced by but-2’FL-OVA-IEC/DC/T cells (Figure 2L) as compared to OVA-IEC/DC/T cells, similar to 2’FL-OVA-IEC/DC/T cells (Table 1). However, the significant increase in IL10 concentration in 3FL-OVA-IEC/DC/T cells (Table 1) was not present in but-3FL-OVA-IEC/DC/T cells, and similar outcomes were found in the absence of OVA (Figure 2L).

The observed differences in T cell functionality could affect subsequent B cell responses, which were not assessed in this *in vitro* model. Furthermore, the immunomodulatory effects of HMOs and SCFAs may be different *in vivo* compared to this *in vitro* model. Therefore, the allergy preventive capacity of HMOs was studied in a murine model of OVA-induced food allergy. *In vivo,* these HMOs can be fermented by the microbiome, yielding SCFAs. Thus, *in vivo* studies into the immunomodulatory effects of HMOs show the combined direct and indirect (via, e.g., the microbiome) effects.

### 3.3 OVA-allergic mice receiving 2’FL or 3FL supplemented diets have altered immunoglobulin levels in serum

To further investigate the effects 2’FL and 3FL may have *in vivo* on allergy development, a murine model for OVA-induced food allergy was used. Mice received HMOs supplemented diets starting 2 weeks before sensitization to OVA and during the entire experiment (Figure 3A). Significant anaphylactic shock and ear swelling were observed in OVA-allergic mice 1 h after intradermal challenge (Figures 3B,C). In addition, OVA-specific immunoglobulin levels in serum were observed in OVA-allergic mice 18 h after oral challenge compared to non-allergic mice (Figures 3D–F). None of the HMOs supplemented diets impacted the allergic shock symptoms or OVA-specific IgE levels in serum (Figure 3D). However, mice receiving 0.5% 3FL diet tended to have a higher OVA-specific IgG1 level (*p* = 0.0837) in serum compared to OVA-allergic mice receiving control diets (Figure 3E). In contrast, mice receiving a 0.1% 2’FL supplemented diet showed significantly lower levels of OVA-specific IgG2a present in serum (Figure 3F) compared to mice receiving control diets. This impacts B cell development during OVA sensitization, potentially modulating the allergic outcomes.

### 3.4 OVA-allergic mice receiving HMO-supplemented diets have altered T cell populations present in MLN

**Figure 4:**
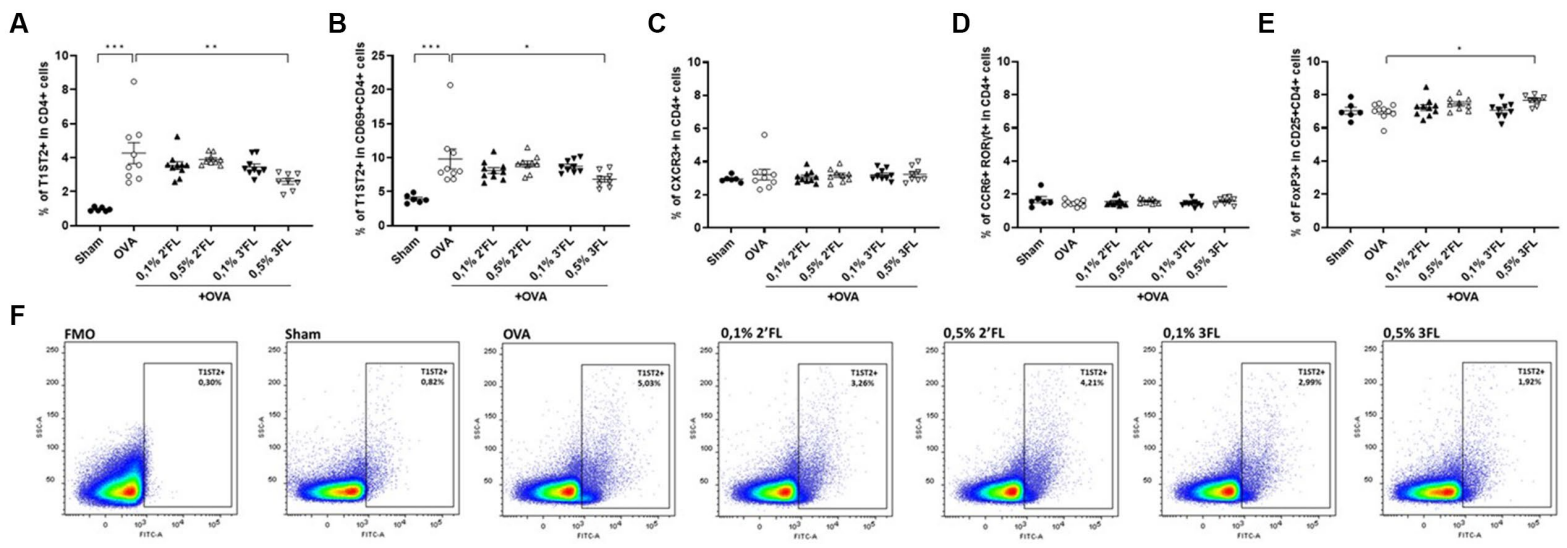
Frequency of T helper subsets in MLNs. The percentage of **(A)** CRTH2+ (Th2), **(B)** CRTH2+ in CD69+ (activated Th2), **(C)** CXCR3+ (Th1), **(D)** CCR6+ RORγt+ (Th17) and **(E)** FoxP3+ in CD25+ (regulatory T) CD4+ cells was measured in MLNs 18 h after oral challenge. **(F)** Representative plots of the CRTH2 gating strategy are shown. Sham and OVA groups were analyzed by an unpaired t-test. All dietary intervention groups were compared to the OVA group using a one-way ANOVA followed by Dunnett’s post-hoc test, *n* = 6–12, mean ± SEM (*p < 0.05, **p < 0.01, ***p < 0.001).

To study the T cell development, which guides B cell development and antibody production, mesenteric lymph nodes (MLNs) were collected 18 h after oral challenge, and local Th subsets were characterized by flow cytometry. Figure 4F shows a representative sample of T1ST2 in CD4+ cells from each experimental group and corresponding FMO control. The proportion of Th2 (T1ST2+ in CD4+ cells, Figure 4A) and activated Th2 cells (T1ST2+ in CD69 + CD4+ cells, Figure 4B) were enhanced in OVA-allergic mice, and percentages of Th1 cells (CXCR3+ in CD4+ cells, Figure 4C), Th17 cells (CCR6 + RORγt+ in CD4+, Figure 4D), and Tregs (FoxP3+ in CD25 + CD4+, Figure 4E) were not affected in OVA-allergic mice. MLNs from mice receiving 0.5% 3FL diets contained a lower percentage of Th2 and activated Th2 cells, with a minor yet significant increase in the Treg population compared to OVA-allergic mice.

### 3.5 HMOs supplemented diets impact mMCP1 concentration in serum and SCFAs levels in cecum content of OVA-sensitized mice

**Figure 5:**
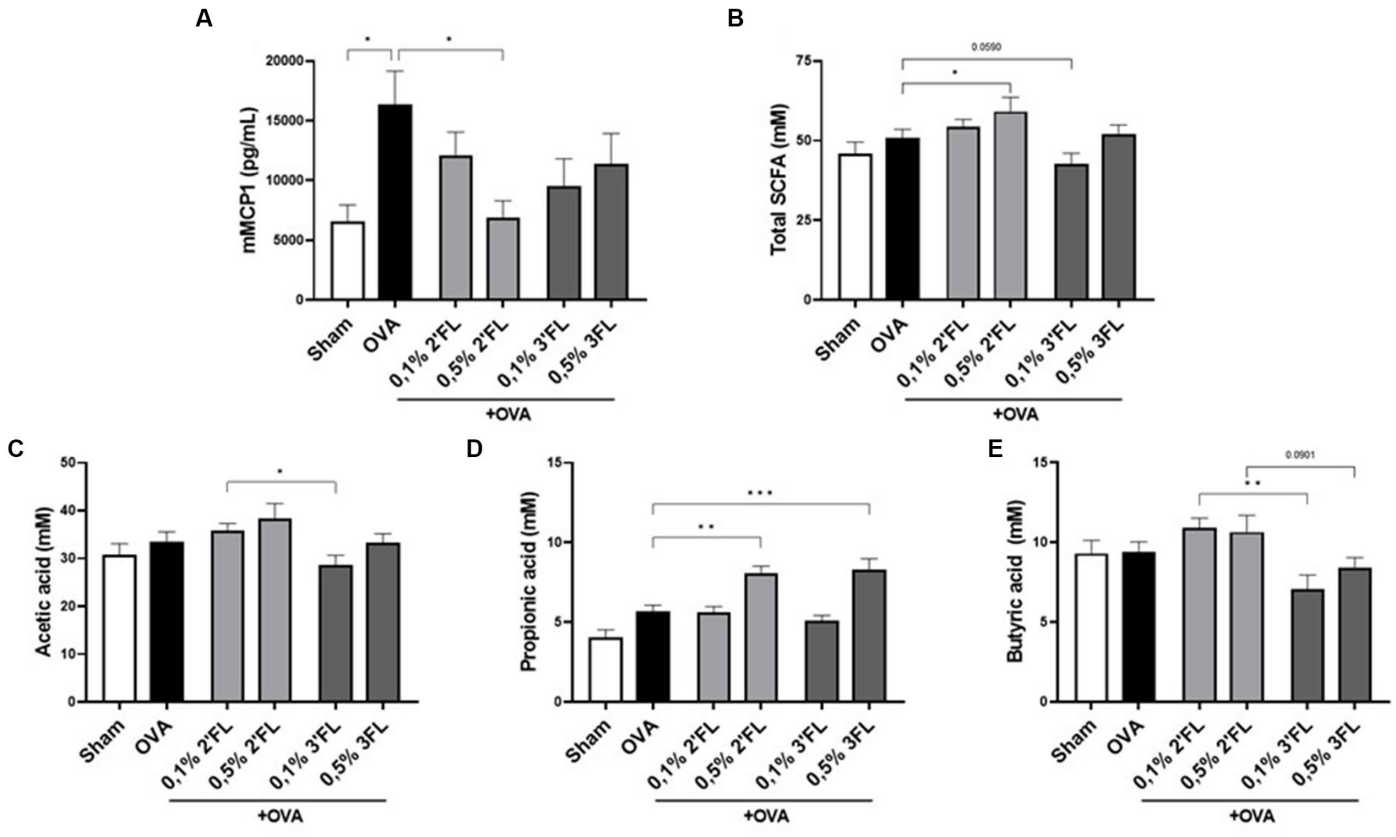
Mucosal mast cell degranulation and presence of SCFAs in cecum content. **(A)** mMCP1 was measured in serum as marker for mucosal mast cell degranulation. **(B)** Total SCFAs, **(C)** acetic acid (acetate), **(D)** propionic acid (propionate), and **(E)** butyric acid (butyrate) levels were determined in cecum content. Sham and OVA groups were analyzed by an unpaired *t*-test (only for mMPC1). All dietary intervention groups were compared to the OVA group using a one-way ANOVA followed by Dunnett’s post-hoc test, *n* = 6–12, mean ± SEM (*p < 0.05, **p < 0.01, ***p < 0.001).

As a marker for mucosal mast cell degranulation after allergen exposure, mMCP1 levels were measured in serum. As expected, the mMCP1 concentration in serum was increased in OVA-allergic mice. Interestingly, although no impact was on detected allergy symptoms such as shock or ear swelling, in mice receiving the 0.5% 2’FL diet, a significantly lower level of mMCP1 was detected, reaching levels observed in the non-allergic mice (Figure 5A). As mast cell degranulation can be affected by HDAC inhibitors, including some SCFAs (22), SCFA levels were measured in the cecum content of allergic and diet-supplemented mice. The mice receiving 0.5% 2’FL diets had significantly higher total SCFA levels (incl. acetate, propionate, butyrate, iso-butyrate, valeric acid, and iso-valeric acid) in their cecum content (Figure 5B), while mice receiving 0.1% 3FL diets had total SCFA levels in cecum content lower as compared to OVA-allergic mice on control diet (*p* = 0.0590). Focusing on the most abundantly present SCFAs, mice receiving 0.1% 2’FL diet had higher levels of acetate and butyrate in the cecum content compared to mice receiving a 0.1% 3FL diet (Figures 5C,E). However, in mice receiving 0.5% supplemented diets, higher propionate levels were measured (Figure 5D). These results are indicative of an immunomodulatory interaction between SCFAs and HMOs, but also illustrate the complexity of location-dependent immunity effects in a whole organism.

## 4 Discussion

Infancy is an important stage of life during which immune maturation occurs; this impact immune fitness and resilience throughout life. Exposure to HMOs via breastmilk contributes to maturation of the immune system, e.g., via interactions with IEC, underlying immune cells and fermentation into other bioactive compounds such as SCFAs (27). As both HMOs and SCFAs are abundantly present in early life, we hypothesized a potential interaction between 2’FL and 3FL with SCFAs on the immunomodulatory effects. The interaction between HMOs and butyrate, as the most potent immunomodulator among the SCFAs (20), was explored in an *in vitro* model for OVA-induced epithelial inflammation. Furthermore, the impact of 2’FL and 3FL supplemented diets was explored *in vivo* using a murine model for OVA food allergy, in which both SCFAs and HMOs can affect local mucosal immunity.

The *in vitro* mucosal immune model used in this manuscript was previously developed to study the effects of allergen-induced epithelial inflammation. The subsequent type 2 skewing of the sequential immune activation, as well as immunomodulatory effects of the HMOs 2’FL and 3FL, are demonstrated within this model (18). Here, this coculture model was initially used to study the interaction between butyrate and 2’FL or 3FL in the absence of an inflammatory trigger (Figure 3). 2’FL and 3FL exposure modulated the subsequent epithelial cell cross-talk with moDCs in such a way that secretion of IL13 in 2’FL-IEC/DC/T cells was enhanced, while 3FL-IEC/DC/T cells showed a significant increase in IFNγ, IL17, and IL10, potentially steering away from the Th2 skewed phenotype. This phenomenon is of importance since a Th2-prone immune response is observed in early life (28, 29), and healthy immune maturation shifts the balance toward Th1 and Treg-driven immunity.

Butyrate, produced upon fermentation of HMOs (30), is well known for its interactions with, e.g., colonocytes and small intestine epithelial cells. This interaction occurs via binding to GPR41 and GPR43, or butyrate becomes intracellularly available via MCT-mediated transport and can act as HDAC inhibitor or suppress NF-κB activation (20, 24, 31). Here, we did not observe any effects of butyrate alone in the homeostatic model, so upon IEC exposure to butyrate alone. Previous studies did not observe alterations in epithelial chemokine and cytokine secretion when the cells were exposed to butyrate in the absence of an inflammatory trigger as well (24, 32, 33).

Although human milk contains butyrate at physiologically relevant levels (median concentration of 0.75 mM, depending on, e.g., stage of lactation (34)), the HMOs present in human milk are fermented by specific intestinal bacteria into SCFAs (30) and will therefore naturally be present together in the infant’s intestine. Here, we also observed that combined exposure of butyrate with 2’FL enhanced secretion of hallmark indicator of type 1 immunity IFNγ, and regulatory IL10 in IEC/DC/T cells on top of the 2’FL associated IL13 increase observed in T cells instructed by primed DC from 2’FL-IEC/DC cultures. On the contrary, but-3FL-IEC/DC/T cell supernatants did not contain significantly elevated levels of IL17 and IL10 as was observed for 3FL-IEC/DC/T cells, while the enhanced IFNγ concentration remained present. Indicating a differential effect of butyrate combined with either 2’FL or 3FL in this mucosal immune coculture model, this difference could be explained by, e.g., different receptor interactions observed between 2’FL and 3FL. For example, in TNFα activated FHs74Int epithelial cell cultures, 3FL, but not 2’FL, inhibits IL8 release via shedding the TNF receptor 1 (35), thereby decreasing the number of available receptors on the cell. Furthermore, 2’FL inhibited IL8 release, IL8, and CD14 mRNA expression, while the concentration of soluble CD14 increased in LPS-triggered T84 cells (36). Interestingly, these effects were not observed for 3FL. Although these studies were not performed in an allergy-focused setting, they demonstrate that the isomers 2’FL and 3FL interact differently with epithelial cells and, therefore, potentially also differentially affect the response to allergic triggers.

In combination with OVA as an allergic inflammatory trigger, differential immunomodulatory effect by pre-incubation with 2’FL and 3FL were observed in this mucosal immune model previously (18). A summary of these data is displayed in Table 1. Here, butyrate pre-incubation prevented the OVA-mediated TSLP release from IEC, while the subsequent coculture steps with moDCs and T cells remained unaffected (Figure 2). TSLP is an epithelial-derived alarmin, known for its contributions to type 2 polarization of DCs and subsequent T cells (37), which is also shown in the T cell response downstream of the OVA-IEC/DC culture. Yet, in this current study, even though butyrate prevented OVA-induced TSLP increase, it was insufficient to significantly affect DC and T cell polarization during subsequent coculture steps. Furthermore, butyrate is a long-known suppressor of NF-κB activation (24, 38), which downstream triggers IL8 release. In these *in vitro* models, butyrate was not able to suppress IL8 secretion after OVA exposure and IEC/DC coculture (data not shown).

When butyrate pre-incubation of IEC/DC was combined with either 2’FL or 3FL, similar to the homeostatic conditions without OVA exposure, distinct immunomodulatory effects were observed. A general boost in both inflammatory and regulatory cytokine secretion was observed previously in 2’FL-OVA-IEC/DC/T cells (18), which remained present in but-2’FL-OVA-IEC/DC/T cells, although here type 1 immunity was further promoted. Therefore, although an interaction between butyrate and 2’FL was hypothesized and observed at the level of IEC/DC interaction in this OVA-induced mucosal immune activation model, the effects observed after but-2’FL pre-incubation corresponded to a great extent with the effects observed after 2’FL pre-incubation alone. Nonetheless, combined pre-incubation with butyrate and 3FL enhanced secretion of IL6 and TGFβ significantly for but-3FL-OVA-IEC/DC, which was not observed for the separate butyrate or 3FL pre-incubations. This phenomenon was only observed in the presence of OVA. In the homeostatic model, IL6 secretion remained unaltered by combined 3FL and butyrate exposure, while IL6 increased only in small amounts by 2’FL and butyrate. Previously, it was described that in the presence of IL6, the Treg function is suppressed (39), as well as the production of type 1 IFNγ (40), while the development of type 2 (41) and type 17 response (42) is promoted. However, secretion of IFNγ tended to increase in but-3FL- or 2’FL-OVA-IEC/DC/T cells, which was not observed for the separate butyrate, 3FL, or 2’FL pre-incubation. Interestingly, IL6 and TGFβ together drive the development of a Th subset capable of secreting high levels of both IFNγ and IL17 in mice (43). Indeed, but-3FL or 2’FL OVA-IEC/DC/T cell supernatants contained elevated levels of IL17, which was not the case in the homeostatic model. Thus, butyrate supported 3FL or 2’FL in driving type 1 and type 17 responses in this inflammatory condition. In addition, OVA-induced type 2 immunity was suppressed by 3FL and in combination with butyrate. Although IL6 was increased after 3FL pre-incubation combined with butyrate, other factors may have prevented type 2 development during pre-incubation with 3FL alone. This was not the case for butyrate and 2’FL, but in this condition, IL10 secretion remained elevated, which may also support to counteract type 2 immunity. The initiation of this regulatory response was already shown at the level of the IEC/DC culture where, solely in the condition of 2’FL and butyrate, increased IL10 secretion was observed. IL10 secreted from DCs is required for the development of IL10 producing T cells and has been found to promote individual’s outcomes during allergy immunotherapy (44, 45).

Overall, these *in vitro* studies indicated that 3FL may be capable of suppressing OVA-induced type 2 responses, while enhancing type 1 and 17 immunity. In contrast, 2’FL could not suppress type 2 immune development, but beyond type 1 and type 17, a regulatory response developed in the presence of butyrate. To validate our *in vitro* findings, a murine study was performed. Mice received 2’FL or 3FL supplemented diets 2 weeks prior to the start of sensitization to explore the allergy preventive effects of the supplemented diets. Although clinical symptoms were not alleviated by the dietary interventions, a shift in humoral response was observed. OVA-specific IgE levels were not affected in mice receiving 2’FL or 3FL supplemented diets, yet mice receiving the 0.1% 2’FL diet had lower levels of OVA-specific IgG2a in their serum. Murine IgG2a is functionally compared to human IgG1, which is associated with antiviral responses but can also be produced upon allergen exposure (46). The production of murine IgG2a upon vaccination can be enhanced via dietary 2’FL (47), yet here, a decrease in OVA-specific IgG2a was observed in OVA-allergic mice receiving dietary 2’FL. The modulation of B cell responses by dietary 2’FL therefore seems to be context- or trigger-dependent. Furthermore, mice receiving the 0.5% 3FL diet had higher levels of OVA-specific IgG1 in their serum. Murine IgG1 has been functionally paired with human IgG4 (48), which is considered non-inflammatory, and increased levels are found during successful allergen immunotherapy (46, 49).

As antibody secretion by B cells is regulated by interaction with T helper cells, local T helper subset development was assessed in MLNs (Figure 4). A significantly lower percentage of total Th2 and activated Th2 cells was found in mice receiving 0.5% 3FL diets; furthermore, MLNs from these mice contained a higher proportion of regulatory T cells, while the Th1 and Th17 populations remained unaffected. This shift in local T helper response in mice receiving a 0.5% 3FL diet may be sufficient to explain the observed shift in the humoral response, as the production of regulatory cytokines combined with lower levels of type 2 cytokines has been linked to an enhanced IgG1 production in mice (50).

Binding of allergen-specific IgE to mucosal mast cells is crucial to elicit mast cell degranulation upon subsequent exposure to the allergen. Many bioactive compounds, such as mMCP1, are released during mast cell degranulation that are involved in symptom induction. mMCP1 in the serum of mice receiving the 0.5% 2’FL diet was significantly decreased. Lower levels of mMCP1 in serum tended to correlate with fewer clinical symptoms as measured by the acute skin response (Spearman correlation, *r*^2^ = 0.352, *p* = 0.069); however, no significant effects were observed in reducing clinical symptoms. IgE-crosslinking is essential to induce mast cell degranulation, yet we did not observe significant differences in serum OVA-specific IgE levels in mice receiving a 2’FL or 3FL supplemented diet. However, many other factors are known to regulate mast cell stability, such as the presence of regulatory cytokines (51) or HDAC inhibitors such as butyrate and propionate (22). The latter was further addressed by measuring SCFAs in the cecum content of the mice. Mice receiving the 0.5% 2’FL diet had higher SCFA levels in their cecum content, in association with the observed decrease in mMCP1 levels in serum. mMCP1 is a biomarker of mucosal mast cell activation (52), which may indicate a local protective effect on mast cell degranulation in the intestine. Administration of SCFAs in the drinking water of mice has previously been shown to improve food allergic outcomes, such as lower anaphylaxis scores and serum IgE levels (53). Furthermore, high fecal levels of SCFAs during early life have been linked to a lower incidence of atopic sensitization at 1 year of age (25). However, further substantiating the connection among SCFAs, HMOs, and allergy prevention, as well as the mechanisms of possible interaction between SCFAs and HMOs in immunomodulation, is warranted.

Some of the *in vitro* immunomodulatory effects of butyrate, 2’FL, and 3FL could be observed in the murine study as well. The reduced IL13 secretion by 3FL pre-incubation *in vitro* corresponds to the reduced percentage of Th2 cells observed *in vivo* in this chronic model for food protein sensitization. However, the presented *in vitro* model lacks multiple components which are present in the murine model. For example, this *in vitro* model focuses on the modulation of IEC and the subsequent cross-talk between these IEC and DC, following the effects of these primed DC on the T cells. Beyond possible effects on IEC, in the murine model, butyrate, 2’FL, and 3FL may become systemically available and in direct contact with immune cells. Therefore, they may also directly affect immune cell functioning. In addition, the humoral response and effector response were monitored in the murine model, and these effects were not addressed in the current *in vitro* study. Future improvements of the *in vitro* model should allow interaction between butyrate, 2’FL, and 3FL and moDC and/or T cells to provide a more complete overview of the immunomodulatory properties of these structures. Now, these components were mainly exposed to the IEC. Furthermore, the *in vitro* model should be expanded in future experiments by including B cells and mast cells to assess the complete immunological cascade involved in allergic sensitization and effector phase (54).

Even though the HMOs did show beneficial effects on allergy biomarkers in the murine model for ovalbumin-induced food allergy, the allergic sensitization (OVA-IgE) and systemic allergic symptoms scores were not prevented. The dosing of HMOs in the diets was based on the physiological relevant range observed in human milk. Human milk contains 5–20 g/L HMOs (0,5–2%), depending on the stage of lactation and several other factors. 2’FL and/or 3FL are generally the most abundantly present HMOs in human milk (55). However, in future studies, other doses of HMOs, or alternatively combinations of HMOs, may be studied for their allergy preventive effects. In addition, a combination of HMOs and a beneficial bacterial strain may be considered. Previously, our group had shown the additive beneficial effect when dietary non-digestible oligosaccharides were combined with *Bifidobacterium breve* M16V in the prevention of cow’s milk allergy in mice (56).

## 5 Conclusion

The present study investigated two common fucosylated human milk oligosaccharides, 2’FL and 3FL, for their impact on allergy-related responses in an *in vitro* mucosal immune model and an *in vivo* model for hen’s egg (ovalbumin) allergy. *In vitro* butyrate exposure promoted the development of a downstream type 1 and regulatory response in the presence of 2’FL in homeostasis but did not affect immunomodulatory effects of 3FL. 2’FL and 3FL differentially modulated ovalbumin-induced mucosal inflammation independent of the presence of butyrate. Dietary supplementation with 3FL lowered Th2 frequency while enhancing Treg, but both 2’FL and 3FL diets did not affect food allergic symptoms or OVA-specific IgE within this murine model. 2’FL, however, improved the humoral immune response and lowered mucosal mast cell activation in association with increased fecal SCFA levels. Moreover, these results indicate that the interaction as present *in vivo* between effects of SCFAs and HMOs are important to understand immune development *in vivo*.

## Data availability statement

The raw data supporting the conclusions of this article will be made available by the authors, without undue reservation.

## Ethics statement

Ethical approval was not required for the studies involving humans because human blood was obtained for healthy volunteers via the national blood bank. The studies were conducted in accordance with the local legislation and institutional requirements. The human samples used in this study were acquired from healthy volunteers via the national blood bank. Written informed consent to participate in this study was not required from the participants or the participants’ legal guardians/next of kin in accordance with the national legislation and the institutional requirements.

## Author contributions

MZ: Conceptualization, Investigation, Writing – original draft. MD: Investigation, Writing – review & editing. PK: Investigation, Writing – review & editing. JG: Supervision, Writing – review & editing. GF: Supervision, Writing – review & editing. BL: Conceptualization, Supervision, Writing – review & editing. LW: Conceptualization, Supervision, Writing – review & editing.

## Glossary

2’FL: 2′-fucosyllactose
2’FL-IEC/DC: Dendritic cells that were cocultured with intestinal epithelial cells exposed to 2′-fucosyllactose
2’FL-IEC/DC/T cell: T cells which were cocultured with dendritic cells that were cocultured with intestinal epithelial cells exposed to 2′-fucosyllactose
2’FL-OVA-IEC/DC: Dendritic cells that were cocultured with intestinal epithelial cells exposed to 2′-fucosyllactose prior to and during ovalbumin stimulation
2’FL-OVA-IEC/DC/T cell: T cells which were cocultured with dendritic cells that were cocultured with intestinal epithelial cells exposed to 2′-fucosyllactose prior to and during ovalbumin stimulation
3FL: 3-fucosyllactose
3FL-IEC/DC/T cell: T cells which were cocultured with dendritic cells that were cocultured with intestinal epithelial cells exposed to 3-fucosyllactose
3FL-OVA-IEC/DC/T cell: T cells which were cocultured with dendritic cells that were cocultured with intestinal epithelial cells exposed to 3-fucosyllactose prior to and during ovalbumin stimulation
ANOVA: Analysis of variance
but-2’FL-IEC/DC: Dendritic cells that were cocultured with intestinal epithelial cells exposed to 2′-fucosyllactose and butyrate
but-2’FL-OVA-IEC/DC: Dendritic cells that were cocultured with intestinal epithelial cells exposed to 2′-fucosyllactose and butyrate prior to and during ovalbumin stimulation
but-3FL-IEC: Intestinal epithelial cells exposed to 3-fucosyllactose and butyrate
but-3FL-IEC/DC: Dendritic cells that were cocultured with intestinal epithelial cells exposed to 3-fucosyllactose and butyrate
but-3FL-OVA-IEC/DC: Dendritic cells that were cocultured with intestinal epithelial cells exposed to 3-fucosyllactose and butyrate prior to and during ovalbumin stimulation
but-IEC: Intestinal epithelial cells exposed to butyrate
but-OVA-IEC: Intestinal epithelial cells exposed to butyrate prior to and during ovalbumin stimulation
but-OVA-IEC/DC-T cell: T cells which were cocultured with dendritic cells that were cocultured with intestinal epithelial cells exposed to butyrate prior to and during ovalbumin stimulation
CCL: C-C motif ligand
DC: Dendritic cell
GPR: G-protein coupledceptor
HDAC: Histone deacetylase
HMOs: Human milk oligosaccharides
IEC: Intestinal epithelial cell
IEC/DC/T cell: T cells which were coculture with dendritic cells that were previously cocultured with intestinal epithelial cells
IFNγ: Interferon γ
Ig: Immunoglobulin
IL: Interleukin
LPS: Lipopolysaccharide
MCT: monocarboxylate transporter
MLN: Mesenteric lymph node
moDC: Monocyte-derived dendritic cell
NDO: Non-digestible oligosaccharide
NF-κB: Nuclear factor kappa-light-chain-enhancer activator of B cells
OVA: Ovalbumin
OVA-IEC: Intestinal epithelial cells apically exposed to ovalbumin
OVA-IEC/DC: Dendritic cells that were cocultured with intestinal epithelial cells that were apically exposed to ovalbumin
PBMC: Peripheral blood mononuclear cells
RALDH: Retinal dehydrogenase
SCFAs: Short-chain fatty acids
TGFβ: Transforming growth factor β
Th cell: T helper cell
Treg cell: Regulatory T cell
TSLP: Thymic stromal lymphopoietin
