# Supplementary material for: Butyrate interacts with the effects of 2’FL and 3FL to modulate in vitro ovalbumin-induced immune activation, and 2’FL lowers mucosal mast cell activation in a preclinical model for hen’s egg allergy
Source: Front Nutr. 2023 Dec 19;10:1305833. doi: 10.3389/fnut.2023.1305833 (PMC10762782; doi:10.3389/fnut.2023.1305833)
Supplement: Supplementary file 1 [file Data_Sheet_1.docx]

Supplementary Material

# Supplementary Figures


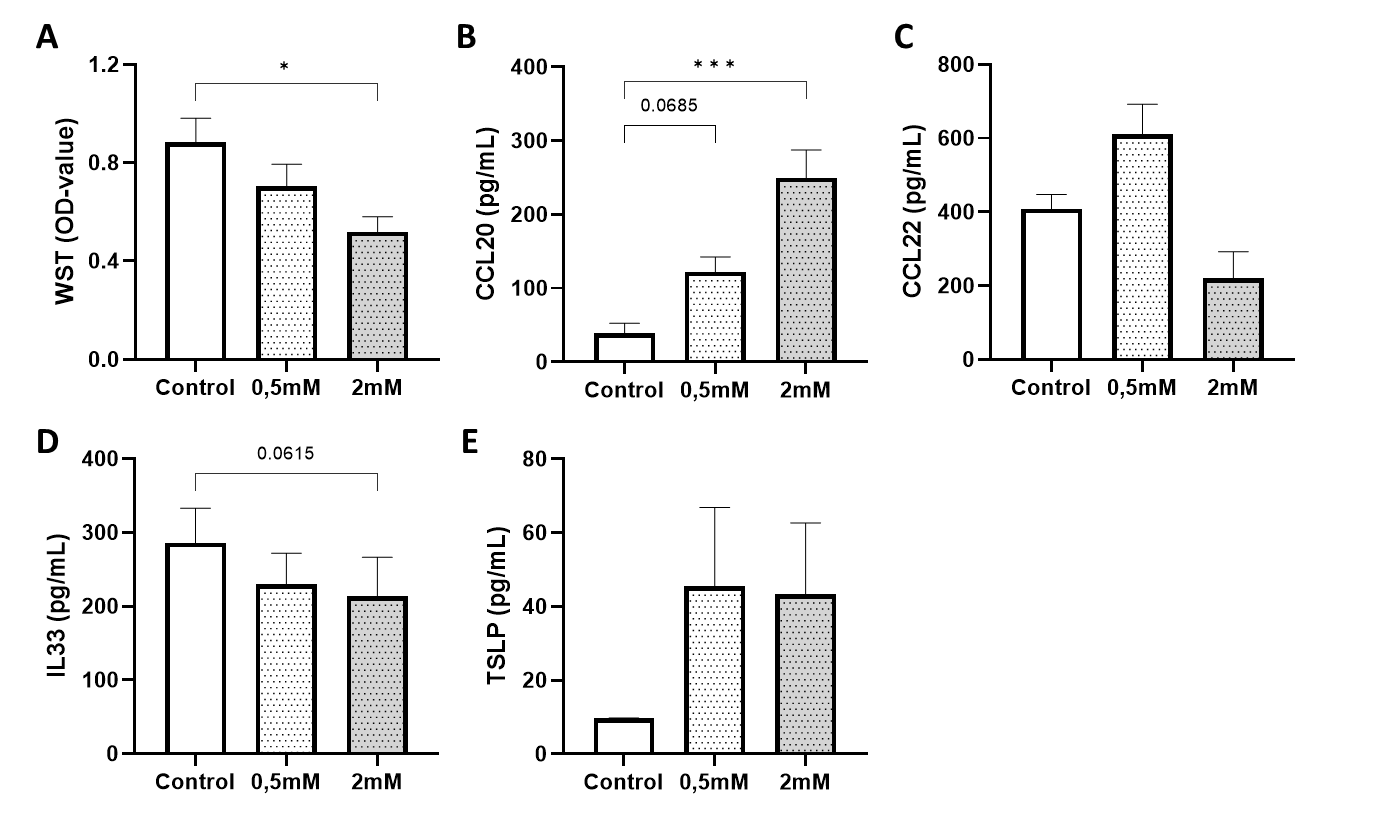


**Supplemental Figure 1.** Dose of butyrate for *in vitro* use was determined by exposing 5 different passages of IEC to butyrate in a 48 wells plate, Confluent HT29 cells were exposed to 0.5 or 2mM butyrate for 48h. After 48h, a) a WST-assay was performed to assess mitochondrial activity as a measure of viability, showing that 2mM butyrate significantly decreased cell viability. Secretion of the chemokines b) CCL20 and c) CCL22 as well as secretion of the alarmins d) IL33 and e) TSLP was measured. Data is analyzed by One-Way ANOVA followed by Dunett’s post hoc test, n=5, mean ± SEM (*p < 0.5, **p < 0.01, ***p < 0.001).


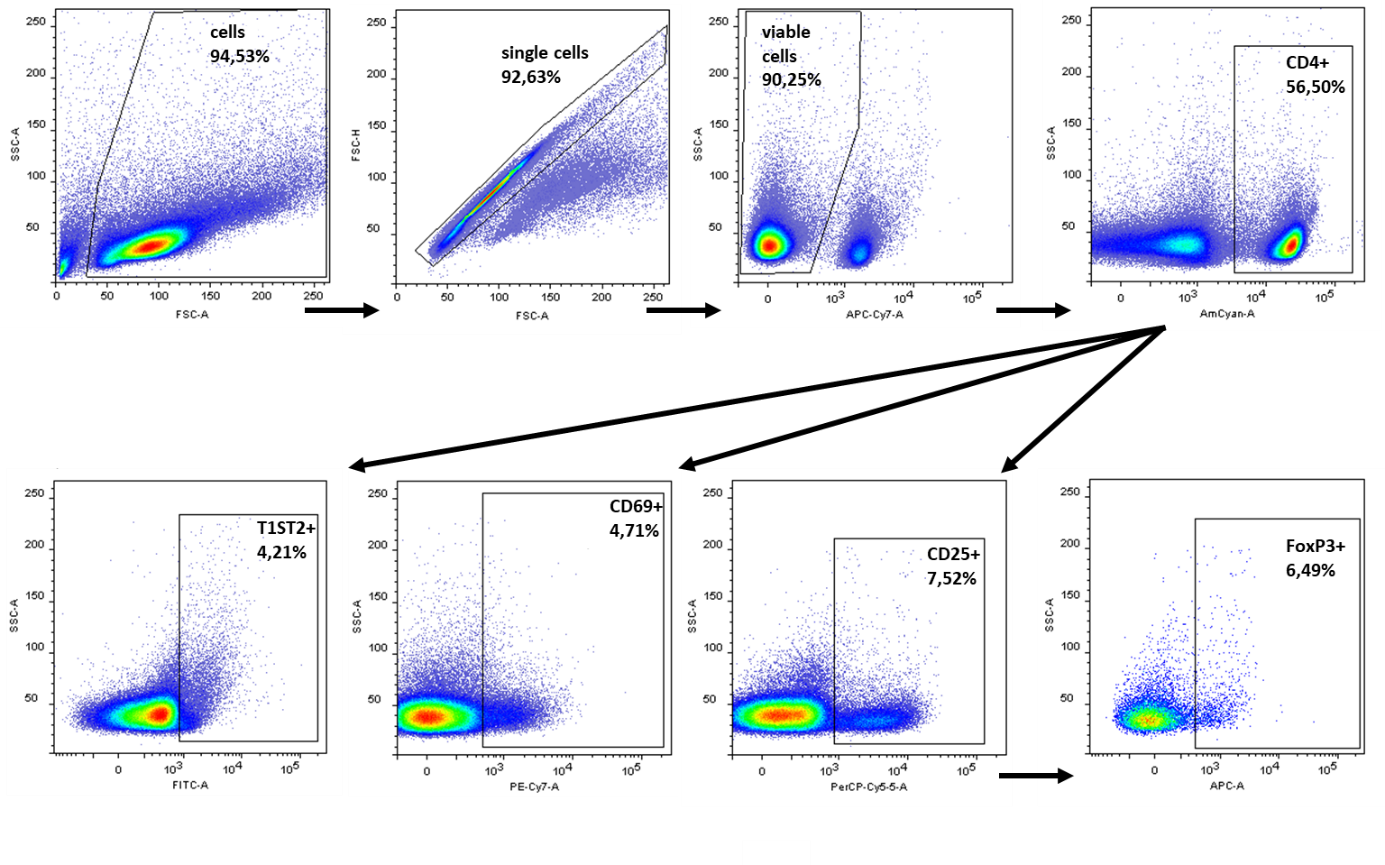


**Supplemental Figure 2.** A representative gating strategy is shown to determine T cell subset populations in murine MLN samples. Appropriate FMO controls were used.

**Supplemental Figure 3** Heatmap, based on z-scores, of the *in vitro* markers measured. Control and OVA exposed conditions as well as preincubations with butyrate and/or A) 2’FL or B) 3FL are presented.
